# Disrupted Network Topology Contributed to Spatial Navigation Impairment in Patients With Mild Cognitive Impairment

**DOI:** 10.3389/fnagi.2021.630677

**Published:** 2021-06-03

**Authors:** Weiping Li, Hui Zhao, Zhao Qing, Zuzana Nedelska, Sichu Wu, Jiaming Lu, Wenbo Wu, Zhenyu Yin, Jakub Hort, Yun Xu, Bing Zhang

**Affiliations:** ^1^Department of Radiology, The Affiliated Drum Tower Hospital of Nanjing University Medical School, Nanjing, China; ^2^Department of Neurology, The Affiliated Drum Tower Hospital of Nanjing University Medical School, Nanjing, China; ^3^Department of Neurology, The Czech Brain Ageing Study, Memory Clinic, Second Faculty of Medicine–Charles University, University Hospital in Motol, Prague, Czechia; ^4^International Clinical Research Center, St. Anne’s University Hospital Brno, Brno, Czechia; ^5^Department of Geriatrics, The Affiliated Drum Tower Hospital of Nanjing University Medical School, Nanjing, China

**Keywords:** graph theory, clustering coefficient, spatial navigation, mild cognitive impairment, network topology

## Abstract

Impairment in spatial navigation (SN) and structural network topology is not limited to patients with Alzheimer’s disease (AD) dementia and can be detected earlier in patients with mild cognitive impairment (MCI). We recruited 32 MCI patients (65.91 ± 11.33 years old) and 28 normal cognition patients (NC; 69.68 ± 10.79 years old), all of whom underwent a computer-based battery of SN tests evaluating egocentric, allocentric, and mixed SN strategies and diffusion-weighted and T_1_-weighted Magnetic Resonance Imaging (MRI). To evaluate the topological features of the structural connectivity network, we calculated its measures such as the global efficiency, local efficiency, clustering coefficient, and shortest path length with GRETNA. We determined the correlation between SN accuracy and network topological properties. Compared to NC, MCI subjects demonstrated a lower egocentric navigation accuracy. Compared with NC, MCI subjects showed significantly decreased clustering coefficients in the left middle frontal gyrus, right rectus, right superior parietal gyrus, and right inferior parietal gyrus and decreased shortest path length in the left paracentral lobule. We observed significant positive correlations of the shortest path length in the left paracentral lobule with both the mixed allocentric–egocentric and the allocentric accuracy measured by the average total errors. A decreased clustering coefficient in the right inferior parietal gyrus was associated with a larger allocentric navigation error. White matter hyperintensities (WMH) did not affect the correlation between network properties and SN accuracy. This study demonstrated that structural connectivity network abnormalities, especially in the frontal and parietal gyri, are associated with a lower SN accuracy, independently of WMH, providing a new insight into the brain mechanisms associated with SN impairment in MCI.

## Introduction

There is growing evidence that the human brain is a large-scale complex network (Seeley et al., 2009; Betzel et al., 2012). A network is represented as nodes that are connected by edges in the graph theory. A node is a brain region, and an edge is constructed by anatomical tracts or functional correlations. The structural network topological properties include the network efficiency properties (local efficiency and global efficiency), clustering coefficient (Cp), local shortest path length (Lp), and the node degree and betweenness centrality for the node properties. The global efficiency of a network (Eg) is measured by how information is exchanged over the network, meaning that how efficient the communication is between one brain region to another, and the local efficiency (Eloc) reflects the average efficiency of each local cluster of the network. Cp reflects that regions that are connected to the same region tend to be also connected to each other. Lp describes how many steps is needed for one brain region to be connected to another. The detailed definition of these properties coincided with several previous studies (Watts and Strogatz, 1998; Latora and Marchiori, 2001). The white matter structural networks in the healthy human brain usually exhibit a small-world character, which can optimally balance information segregation and integration, resulting in efficient organization that not only reduces the cost of maintaining many connections but also allows for efficient information movement (Gong et al., 2009). In contrast, patients with Alzheimer’s disease (AD) dementia and mild cognitive impairment (MCI) showed abnormal properties of cortical networks and loss of small-world characteristics in previous studies that reported either local or global structural connectivity disruptions in these patients (Shu et al., 2012).

Spatial navigation (SN) is a complex domain that refers to the process of determining and maintaining a trajectory from one place to another (Lithfous et al., 2013). Specifically, there are two basic subtypes of SN: egocentric navigation (body-centered) and allocentric navigation (world-centered) (Nedelska et al., 2012). Impairment in both subtypes of SN is frequently reported in both AD dementia and MCI patients. Previous studies have indicated that these SN impairments are related to the degeneration in several brain regions, such as the hippocampus, caudate nucleus, and medial temporal lobe (Wegman et al., 2014). However, given the complexity of the human brain SN system, the structural connectivity networks that integrate these regions may also play critical roles in the SN process, and it would be beneficial to investigate their possible impairment in AD dementia and MCI. However, to the best of our knowledge, few studies have investigated the influence of structural network topological properties on SN.

In this study, we aimed to identify (1) which structural network topological properties show the greatest differences between MCI patients and normal controls (NCs) and (2) how these network properties of specific brain regions affect egocentric and allocentric SN accuracy in MCIs. We hypothesized that patients with MCI would demonstrate abnormalities in brain network topology and that these topological properties (e.g., global efficiency, clustering coefficient, and shortest path length) derived from the brain structural network could influence SN, which might provide a new insight into the structural basis of SN in the brain.

## Materials and Methods

### Subjects

A total of 60 participants, 32 MCI patients and 28 NCs, were recruited from the Department of Neurology of the Affiliated Drum Tower Hospital of Nanjing University Medical School from May 2015 to June 2017. All subjects gave written informed consent to participate in the study, which was approved by the hospital ethics committee.

Exclusion criteria for NCs were the presence of cognitive complaints and neurological or psychiatric disorders. All participants were right-handed and underwent neuropsychological tests, including the Mini-Mental State Examination (MMSE) and Montreal Cognitive Assessment (MoCA). Patients with MCI met the clinical criteria established by Petersen (2004). The threshold for memory impairment was derived from the same literature and designated as scoring > 1.5 SD below the mean of age- and education-adjusted norms on a memory test.

### Spatial Navigation Tests

Spatial navigation accuracy was tested by the PC test AMUNET (NeuroScios GmbH, Austria) that represents a human analog of the Morris water maze (MWM) task screen, which used the hidden goal task similar to previous studies (Weniger et al., 2009; Nedelska et al., 2012; Laczó et al., 2014). It is designed to distinguish two different strategies of navigation, egocentric (“Ego”) representations concerning self-centered navigation and encoding spatial information from the viewpoint of the navigator, whereas allocentric (“Allo”) strategies are centered on the object rather than on the observer (Lithfous et al., 2013). The AMUNET SN test battery was administered using three SN subtasks. Each subtask involved eight trials, hence 24 trials all together. The tasks were performed in a fixed order with increasing demanded. First, the Allo–Ego mixed subtask was first performed. A large circle representing the overhead view of the task arena was shown on the screen (280 pixels in diameter on a screen with a resolution of 640 × 480 pixels) (Qing et al., 2017). Participants were required to locate the goal point using its spatial relationship with both the starting position and the two distal orientation cues on the circle. Next, the Ego subtask was performed, which required the participants to use only the starting position to locate the goal when distal orientation cues were not displayed. Finally, in the Allo subtask, the participant was only allowed to use solely two distal orientation cues on the arena wall during SN to the goal, whereas the start position was randomly regenerated in each trial and was therefore unrelated to the correct goal position. The positions of the goal point were stable relative to (1) the positions of the starting location and orientation cues in the mixed Allo–Ego subtask, (2) the positions of the start location in the Ego subtask, and (3) the positions of orientation cues in the Allo task. The accuracy of the task was automatically recorded as the distance error between the participants’ final position and the actual goal location in millimeters. The SN performance from eight attempts per each subtask was averaged into the average total error per task (Chen et al., 2021). The time was unlimited to avoid the effect of individual differences in sensory and physical functioning.

### MRI Techniques

Whole-brain MRI scans were obtained using an eight-channel phased array coil (Achieva 3.0T TX, Philips Medical Systems, Best, Netherlands). A three-dimensional high-resolution sagittal T_1_W with turbo fast echo (3D-T_1_TFE) acquisition was performed with a repetition time (TR), echo time (TE), and inversion time (TI) of 9.8, 4.6, and 900 ms, respectively. The other acquisition parameters were as follows: flip angle, 8°; matrix size, 256 × 256; field of view (FOV), 256 × 256 × 256 mm; isotropic resolution, 1.0 mm; slices in the third dimension, 192; and acquisition time, 6 min 43 s. Diffusion tensor imaging (DTI) was encoded along 32 independent orientations, and the *b*-value was 1,000 s/mm^2^. The imaging parameters were as follows: TR/TE, 9,154/55 ms; FOV, 224 × 224 mm; slice thickness 2.5 mm; voxel size 2 × 2 × 2.5 mm^3^; and acquisition time, 6 min 27 s.

### Network Node and Edge Definition

We used the AAL atlas to parcellate the whole brain into 90 areas (45 regions in each hemisphere), which were defined as the nodes of the brain graph. The AAL atlas was transformed from Montreal Neurological Institute (MNI) space to T1 native space, which was non-linearly registered from the individual T1-weighted images.

The pre-processing of DTI data was carried out by PANDA (a pipeline toolbox for analyzing brain diffusion images) (Cui et al., 2013). The main procedure of PANDA includes (1) converting DICOM files into Neuroimaging Informatics Technology Initiative (NIfTI) imaging, (2) estimating the brain mask by using the bet command of the FMRIB Software Library (FSL), (3) cropping the raw images to cut off non-brain space in the raw images, and (4) correcting for the eddy-current effect by using the flirt and the eddy-correct FSL commands.

Then, we used PANDA to perform deterministic fiber tracking to obtain the fractional anisotropy (FA) matrix in two steps: (1) two nodes (regions) were considered to be structurally connected by an edge when the FA value of fiber tracts located in these two regions were between 0.2 and 1, and then (2) weighted structural networks represented by symmetric 90 × 90 matrices were constructed for each individual.

### Network Parameter Analysis

Graph theoretical analysis was performed on the interregional connectivity matrix by using GRETNA^1^, a graph theoretical network analysis toolbox for imaging connectomics. The weighted network properties were calculated, with a sparsity range of 0.05–0.4 with a step size of 0.01. Sparsity was defined as the total number of edges divided by the maximum possible number of edges. Because there is no gold standard to select a single threshold, we calculated the parameters with different thresholds. Finally, the networks were constructed at the sparsity of 0.14, which ensured all nodes included in the networks to present the nodal characteristics of the networks and ensured the most characteristic small-world topology. GRETNA was used to calculate the structural network topological properties, including the network efficiency properties (local efficiency and global efficiency), local Cp, global clustering coefficient [M(Cp)], local shortest path length (Lp), global shortest path length [M(Lp)], and the node degree and betweenness centrality for the node properties. For each subject, 1,000 times randomization was applied, and each time a corresponding random network was generated. Then, the random distribution of Cp and Lp was used to transform real Cp and Lp into a *Z* score by their position in the random distribution as previous studies (Wang et al., 2015). The brain networks were visualized with BrainNet Viewer^2^ (Xia et al., 2013).

### Measurement of WMH Volume

The total volume of white matter hyperintensity (WMH) on 3D-FLAIR images was automatically detected and quantified using the Wisconsin White Matter Hyperintensities Segmentation Toolbox (W2 MHS), which is an open-source toolbox. The major steps involved in WMH volume detection and measurement are as follows: (1) a pre-processing module in which SPM12b was used to construct the white matter (WM) region of interest and partial volume estimates of the tissues (WM, gray matter, and cerebrospinal fluid); (2) a segmentation module in which the random forest-based regression method was used to detect the WMH; and (3) a quantification module to summarize the WMH segmentations.

### Statistical Analysis

Statistical analysis was performed using SPSS version 23.0 for the demographic data. The between-group differences of whole-network and nodal properties and differences in SN accuracy by average total error in each navigational subtask were evaluated by two-sample *t*-tests using a threshold of *p* < 0.05. For each whole-network topological property showing a significant difference between MCI patients and NCs, a general linear regression analysis was performed using two linear models between each of the network properties with the SN accuracy of each subtask. In model 1, the network property was used as an independent variable, and SN accuracy was used as a dependent variable, with age, sex, and education as covariates. In model 2, WMH volume was additionally included as an independent variable. We used a statistical significance level of *p* < 0.05 for all these analyses. Similarly, for each node showing significantly different nodal topological properties between MCI patients and NCs, the same correlational analyses were performed between the corresponding property and the accuracy of each of the three SN subtasks with the same covariates.

## Results

### Demographics and Behavioral Data

In this study, 32 subjects fulfilled the criteria of MCI. No significant differences in age (*p* = 0.194), sex (*p* = 0.196), or education level (*p* = 0.134) were detected between MCI patients and NCs. As expected, pathological alteration led to significant differences in the MMSE and MoCA scores between MCI patients and NCs. The full demographic and clinical characteristics of the subjects are shown in Table 1.

**TABLE 1:** Demographic and clinical characteristics of patients with mild cognitive impairment (MCI) and control participants.

	**MCI (*n* = 32)**	**NCs (*n* = 28)**	***p***
Age (years)			
Mean ± SD	65.91 ± 11.33	69.68 ± 10.79	0.194
Sex (%)			
Male	16 (50%)	19 (67.9%)	0.196
Female	16 (50%)	9 (32.1%)	
Edu (years)			
Mean ± SD	13.25 ± 3.46	14.54 ± 3.05	0.134
WMH (volume, mm^3^)			
Mean ± SD	35,017 ± 37,275	38,850 ± 39,794	0.702
MMSE (score)			
Mean ± SD	25.97 ± 2.36	28.93 ± 0.97	<0.001*
MoCA (score)			
Mean ± SD	21.81 ± 2.13	27.43 ± 2.36	<0.001*

Data are presented as the means ± standard deviations. *p < 0.05.

*MCI, mild cognitive impairment; NCs, normal controls; Edu, education; WMH, white matter hyperintensity; MMSE, Mini-Mental State Examination; MoCA, Montreal Cognitive Assessment, Beijing Version.*

### Spatial Navigation and Network Topology Properties

**FIGURE 1:**
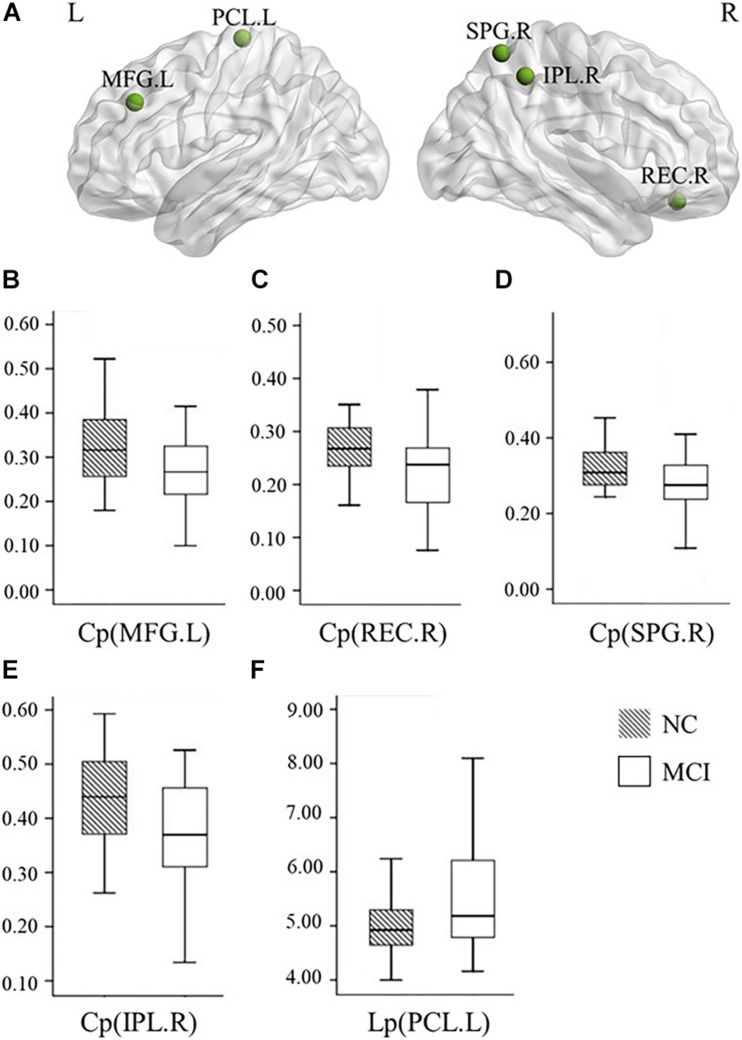
Graphs show differences in the nodal network topological properties between the patients with MCI and normal controls. **(A)** The location of the node with significantly altered nodal network topological properties in MCI patients, compared with normal controls. **(B–E)** The nodal clustering coefficients in the left MFG (*p* = 0.020), right REC (*p* = 0.023), right SPG (*p* = 0.034), and right IPL (*p* = 0.009) were significantly different between the patients with MCI and normal controls. **(F)** The nodal shortest path length in the left PCL (*p* = 0.045) was significantly different between the patients with MCI and normal controls. NC, normal controls; MCI, mild cognitive impairment; L, left; R, right; Cp, local clustering coefficient; Lp, local shortest path length; MFG, middle frontal gyrus; REC, rectus; SPG, superior parietal gyrus; IPL, inferior parietal gyrus; PCL, paracentral lobule.

Our statistical analyses showed significant decreases in the global clustering coefficient and shortest path length in patients with MCI (Table 2). Regarding SN accuracy, MCI subjects showed worse Ego navigation accuracy compared to NCs (Table 2) with larger average total error. The specific areas of discrepant network properties of MCI patients and NCs are listed in Table 3 and Figure 1, including the left middle frontal gyrus, right rectus, right superior parietal gyrus, right inferior parietal gyrus, and left paracentral lobule.

**TABLE 2:** Differences in spatial navigation accuracy and the whole-brain network topology properties of patients with MCI and normal controls.

	**MCI (*n* = 32)**	**NCs (*n* = 28)**	***T***	***p***
AEV (mm)				
Mean ± SD	11.28 ± 9.59	8.67 ± 4.30	−1.32	0.190
EV (mm)				
Mean ± SD	15.79 ± 9.86	9.73 ± 5.39	−2.89	0.004*
AV (mm)				
Mean ± SD	12.55 ± 8.05	10.62 ± 5.76	−1.06	0.295
Eg				
Mean ± SD	0.20 ± 0.03	0.20 ± 0.02	0.12	0.901
Eloc				
Mean ± SD	0.27 ± 0.03	0.28 ± 0.02	1.14	0.114
M(Cp)				
Mean ± SD	28.03 ± 5.69	31.58 ± 7.07	2.15	0.035*
M(Lp)				
Mean ± SD	25.02 ± 7.53	28.91 ± 7.37	2.02	0.048*
Node betweenness				
Mean ± SD	67.59 ± 11.33	67.52 ± 9.84	−0.02	0.981
Node degree				
Mean ± SD	4.35 ± 0.95	4.43 ± 0.83	0.341	0.735

*Data are presented as the means ± standard deviations. The Eg and Eloc are raw data and the M(Cp) and M(Lp) are *z*-score data.*

*MCI, mild cognitive impairment; NCs, normal controls; AEV, “allocentric + egocentric” average total error; EV, egocentric average total error; AV, allocentric average total error; Eg, global efficiency. Eloc, local efficiency; M(Cp), global clustering coefficient; M(Lp), global shortest path length.*

*^∗^*p* < 0.05.*

**TABLE 3:** Nodal network topology properties in patients with MCI and normal controls.

	**MCI (*n* = 32)**	**NCs (*n* = 28)**	***T***	***p***
**Cp**				
L-middle frontal gyrus				
Mean ± SD	0.27 ± 0.07	0.32 ± 0.10	2.393	0.020*
R-rectus				
Mean ± SD	0.22 ± 0.08	0.26 ± 0.04	2.335	0.023*
R-superior parietal gyrus				
Mean ± SD	0.28 ± 0.09	0.33 ± 0.09	2.169	0.034*
R-inferior parietal gyrus				
Mean ± SD	0.37 ± 0.09	0.43 ± 0.08	2.687	0.009*
**Lp**				
L-paracentral lobule				
Mean ± SD	5.55 ± 1.07	5.07 ± 0.86	-2.053	0.045*

*Data are presented as the means ± standard deviations.*

*Cp, local clustering coefficient; Lp, local shortest path length; MCI, mild cognitive impairment; NCs, normal controls.*

*^∗^*p* < 0.05.*

### The Association Between Nodal Network Topology Properties and Spatial Navigation Accuracy

We observed a significantly positive correlation of the shortest path length in the left paracentral lobule with both the Allo–Ego average total error and the Allo average total error. There are no associations between the network topological properties and Ego average total error. A decreased Cp in the right inferior parietal gyrus was associated with a larger average total error in Allo navigation (Table 4).

**TABLE 4:** General linear regression analyses between nodal network topology properties and spatial navigation accuracy.

	**Allo–Ego**				**Ego**				**Allo**			
	**Model 1**		**Model 2**		**Model 1**		**Model 2**		**Model 1**		**Model 2**	
	**Beta (SE)**	***p***	**Beta (SE)**	***p***	**Beta (SE)**	***p***	**Beta (SE)**	***p***	**Beta (SE)**	***p***	**Beta (SE)**	***p***
Cp												
	−0.2	0.10	−0.1	−0.2	0.10	0.2	0.15	0.2	−0.2	0.07	−0.2	0.07
L-middle frontal gyrus	14	3	70	13	6	45	3	36	22	8	22	7
R-rectus	−0.601	0.550	−0.075	0.594	−0.043	0.749	−0.084	0.535	0.123	0.343	0.105	0.432
R-superior parietal gyrus	0.005	0.971	0.007	0.965	−0.145	0.270	−0.156	0.233	0.011	0.929	0.005	0.967
R-inferior parietal	−0.235	0.078	−0.235	0.080	−0.127	0.339	−0.126	0.337	−0.278	0.028*	−0.278	0.029*
Lp												
L-paracentral lobule	0.361	0.011*	0.361	0.011*	0.235	0.099	0.234	0.098	0.348	0.011*	0.348	0.011*

*The spatial navigation average total errors (AEV, EV, and AV) were set as independent variables. The different network topology properties of specific brain areas in MCI patients were set as dependent variables. In all models, we controlled for age, sex, and education and the volume of WMH was added to model 2.*

*Cp, local clustering coefficient; Lp, local shortest path length.*

*^∗^*p* < 0.05.*

Taking the WMH volume into account, we found that the associations of the shortest path length in the left paracentral lobule with both the Allo–Ego average total error and the Allo average total error were the same as the findings for model 1, as was the decreased Cp for the right inferior parietal gyrus and Allo average total error.

## Discussion

This study measured the brain network abnormality, SN, and cognitive impairment in MCI patients. We found a lower egocentric navigation accuracy in MCI patients compared to NCs. We showed an abnormal organization in the structural connectivity networks of MCI patients, reflected by decreased Cp and decreased Lp. The brain areas of abnormal network properties were in the left middle frontal gyrus, right rectus, right superior parietal gyrus, right inferior parietal gyrus, and left paracentral lobule, therefore predominantly in the frontal and parietal gyri. Further, the abnormal network properties were measured in several other brain regions, including the larger shortest path length in the left paracentral lobule and decreased Cp in the right inferior parietal gyrus. These abnormal network properties predicted the SN impairment, irrespective of the white matter hyperintensities.

Egocentric and allocentric navigation strategies involve different neurobiological underpinnings. Generally, allocentric navigation is mainly supported by the hippocampus and parahippocampus (Muller et al., 1996). On the other hand, egocentric navigation is supposed to rely on the parietal lobe and the retrosplenial cortex mostly (Epstein and Ward, 2010; Nemmi et al., 2017). Successful navigation does not rely on one single strategy but requires the ability to switch between and combine the different spatial strategies in a flexible manner (Colombo et al., 2017). In the previous study, the amnestic MCI single-domain patients showed both the allocentric and the egocentric navigation impairment (Hort et al., 2007). Potentially, because we did not classify our MCIs into amnestic versus non-amnestic subtypes, we found egocentric navigation but not allocentric navigation impairment in these MCI patients. It also might be the relatively younger population of MCI patients in our study, which sometimes show hippocampal sparing subtype of AD (Jellinger, 2020).

To date, SN accuracy has not been explored regarding the relationship to whole-brain structural network properties based on the graph theoretical approach. Subjects using an Allo strategy revealed stronger activations in some nearby basal regions (hippocampus and thalamus) (Henke et al., 2003), while Ego navigation has been shown to rely on corticostriatal regions of the brain (Wolbers and Wiener, 2014). A previous study found that the parietal lobe is involved in the dynamic aspects of spatial memory and makes contribution to topographic memory (Berthoz, 1997). We also found a hypoactive brain structural network in the right inferior parietal gyrus is related to worse allocentric navigation skill. Another fMRI study found SN performance-related activation of the inferior parietal cortex, suggesting that this area participates in the encoding of spatial relationships between consecutive landmarks in an egocentric reference frame, defined relative to the observer’s direction when facing the first landmark (Wolbers et al., 2004). A study using structural MRI study showed that the atrophy of the right inferior parietal cortex in amnesic MCI patients was related to the deficits in allocentric and egocentric navigation toward a target in a familiar virtual environment (Weniger et al., 2011).

The graph theory measures reflect how well a region is connected to its neighboring areas and within brain modules, providing important information on the network’s capability for specialized processing within densely interconnected groups of brain regions (Rubinov and Sporns, 2010). Usually, randomly organized networks are characterized by a low Cp (a measure that depicts the connection of immediate neighbors around individual vertices) and a short path length (an index reflecting the overall integration of the network). The small-world network, characterized by a high degree of clustering and a short path length between individual network nodes, has been an attractive model for the description of complex brain networks (Wu et al., 2012). Researchers have found that both anatomical and functional brain networks are small-world networks (Achard and Bullmore, 2007; Bassett and Bullmore, 2016). The brain network topology showed the small-world characteristic in both AD dementia and MCI, but it changed significantly compared to NCs (Liu et al., 2012; Zhao et al., 2012). A previous study also indicated increased short path length in AD and decreased Cp in amnestic MCI (Bai et al., 2012). In the current study, we found a decreased Cp in MCI patients, which is similar to a previous study, indicating worse local communication between the left middle frontal gyrus, the right superior and inferior parietal gyrus, and neighboring areas in the brain, respectively.

The brain topology alterations of specific brain node regions were also observed, in addition to global network changes. We found a decreased short path length of the left paracentral lobule, which means a loss of the number of connections between these structures and other regions of the network. This could be related to WM integrity loss or a disruption of WM fibers connecting these brain areas, which has been previously observed in MCI and AD patients in DTI studies (Chua et al., 2008). The decreased connectivity of the left paracentral lobule to neighboring areas was related to worse SN accuracy and, more importantly, the burden of WMH had no effects on this relationship. This may indicate that the network alteration and the SN impairment were due to degeneration, not ischemic lesion, which may need to be confirmed with a larger sample size in the future.

There were some limitations in our study. First, we had a relatively small sample size in this study. Second, the computerized test based on the MWM paradigm may be a useful tool for the evaluation of SN deficits (Laczo et al., 2014). However, it should be noted that the real-space and computerized two-dimensional versions are not fully interchangeable, as the computerized SN tasks lack proprioceptive feedback that is normally available in real-world navigation tasks and that contribute to successful navigation. Third, in humans spatial cognition evaluation is much more difficult, as navigation in complex real-world environments does not allow experimental control of the tasks, making it difficult to determine the mechanisms that sustain performances. Fourth, we did not analyze the specific structural connection between any two brain nodes and this would be of great significance in the future.

In conclusion, patients with MCI demonstrate abnormalities in brain network topology, and the disruption of these topological properties (e.g., Cp and shortest path length) derived from the brain structural network influences the SN process. These results may fuel future research on the brain structure basis of SN, which can provide new insight into brain mechanisms in SN impairments with network topological properties.

## Data Availability Statement

The datasets presented in this study can be found in online repositories. The name of the repository can be found at: https://pan.baidu.com/s/14bm0x3tikemnFteKQAJcxw with the following password - r6q8.

## Ethics Statement

The studies involving human participants were reviewed and approved by the Ethics Committee of Gulou Hospital affiliated to Nanjing University Medical College (2016–065–01). The patients/participants provided their written informed consent to participate in this study.

## Author Contributions

All authors listed have made substantial, direct and intellectual contribution to the work, and approved it for publication.

## Conflict of Interest

The authors declare that the research was conducted in the absence of any commercial or financial relationships that could be construed as a potential conflict of interest.

## Footnotes

1 https://www.nitrc.org/projects/gretna

2 http://www.nitrc.org/projects/bnv/
